# Exploration of experiences and attitudes associated with lung health promotion among Black males with a history of smoking

**DOI:** 10.22514/jomh.2024.005

**Published:** 2024-01-29

**Authors:** Alicia K. Matthews, Suchanart Inwanna, Dami Oyaluade, Alexis Chappel, Jennifer Akufo, Sage J. Kim, Rohan Jeremiah

**Affiliations:** 1 School of Nursing, Columbia University, New York, NY 10001, USA; 2 College of Nursing, the University of Illinois Chicago, Chicago, IL 60612, USA; 3 Ramathibodi School of Nursing, Faculty of Medicine Ramathibodi Hospital, Mahidol University, 10400 Bangkok, Thailand; 4 Cancer Center, the University of Illinois Hospital, Chicago, IL 60612, USA; 5 Northeastern Illinois University, Chicago, IL 60625, USA; 6 School of Public Health, the University of Illinois Chicago, Chicago, IL 60612, USA

**Keywords:** Tobacco use, Lung cancer screening, Black males, Qualitative, Lung health promotion

## Abstract

To examine knowledge and attitudes about lung health promotion (smoking cessation and lung cancer screening) among Black male smokers in a large Midwestern city in the United States. Semi-structured, in-depth interviews were conducted with 25 study participants. Each interview lasted approximately 45 minutes. Participants also completed a brief (5–10 minutes) survey measuring demographic characteristics, smoking experiences and knowledge and attitudes about lung health promotion activities. Descriptive statistics were used for quantitative data, and deductive thematic analysis for qualitative data analysis. The mean age of study participants was 57.5 years. Eighty-four percent of participants were current smokers, with the majority being daily smokers. Perceived risk for lung cancer was mixed, with 56% of participants endorsing that they considered themselves to be at high or moderate risk and the remaining 44% at low or no risk for lung cancer. Forty percent of participants reported having had a test to check their lungs for cancer. Participants were aware of the health risks associated with smoking but reported limited assistance from providers regarding the receipt of smoking cessation treatments. Awareness of lung cancer screening was limited, but participants expressed openness to screening; however, barriers were anticipated, including costs, fear and a reduced willingness to be screened in the absence of symptoms. Study participants reported limited experiences with lung health promotion activities. Knowledge about the facilitators and barriers can be used to develop health promotion interventions targeting smoking cessation and lung cancer screening.

## 1. Introduction

Smoking prevalence rates in the United States (US) are historically low, with approximately 12.5% of adults reporting current cigarette smoking [1]. Despite the reduction in overall smoking rates, lung cancer remains the leading cause of cancer-related deaths in the US, accounting for 25% of all cancer-specific deaths [2, 3]. Most lung cancer patients are diagnosed with an advanced stage of diagnosis, characterized by a large tumor size that has spread to other organs [4]. Late-stage diagnosis contributes significantly to the high mortality rates associated with lung cancer [5]. The five-year survival rate for late-stage lung cancer patients is only five percent but increases to 57.9% for patients with localized (Stage 1) cancers [3]. Given the high mortality rate, lung cancer risk reduction and early detection remain significant cancer prevention and control priorities [6].

Central to strategies to improve outcomes associated with lung cancer are efforts to reduce racial/ethnic inequities in screening and diagnosis. A consistent body of epidemiological and clinical data shows that the burden associated with lung cancer incidence and mortality is not equally distributed among adults who smoke [7]. In the U.S., Black men have the highest incidence and mortality associated with lung cancer compared to men from other racial/ethnic groups, despite the lower frequency and intensity of smoking in this group across the life course [8–10]. Furthermore, Black smokers, regardless of gender, are more likely to develop lung cancer at an earlier age than White individuals (median age, 67 *vs.* 70 yrs.) and to be diagnosed with advanced-stage disease (53% among Black individuals *vs.* 49% for Whites) [11]. Socioeconomic factors have been linked to racial/ethnic differences in cancer-related outcomes [12, 13]. However, these observed differences in incidence, stage, and mortality exist among Black smokers regardless of socioeconomic factors such as education and income [14]. Increasing engagement in lung cancer screening allows for early detection and treatment for all racial/ethnic groups [15, 16].

The National Lung Screening Trial (NLST) (2011) demonstrated that low-dose helical computed tomography (LDCT) lung cancer screening in older smokers reduced lung cancer mortality by 15–20% due to the detection of treatable lesions [17]. Subsequently, a study in the Netherlands replicated the results of the NLST trial [18]. Based on the results from the NLST trial, the US Preventive Services Task Force (USPSTF) has recommended annual screening with LDCT in older adults with a history of chronic high-frequency smoking [17]. Even though the Centers for Medicare and Medicaid (CMS) and private insurers provide coverage for annual LDCT screening, uptake of LDCT screening remains low among high-risk smokers [19]. Factors influencing LDCT screening rates in the general population include limited access to screening tests and smoking cessation programs, limited patient acceptance, and inconsistent provider knowledge about screening guidelines [20, 21]. In addition, Black smokers are more likely to be unaware of the availability of lung cancer screening, be under-insured, and have lower socioeconomic status, all of which contribute to suboptimal screening rates for lung cancer [22–25]. In addition, structural barriers—poverty, transportation, racism and interpersonal barriers—fear and medical mistrust, further influence lung cancer screening disparities among Black smokers [26, 27].

Given the known health disparities associated with lung cancer, additional research is needed to understand further factors associated with engagement in high-risk groups’ lung health promotion interventions (smoking cessation and lung cancer screening). This is especially important given that best-practice guidelines emphasize the importance of combining lung cancer screening interventions with smoking cessation recommendations and support [17, 19]. Information relevant to lung health promotion interventions—that is focusing on the dual objectives of lung cancer screening and smoking cessation—among Black male smokers remains limited to date. This paper describes a qualitative study that recruited a sample of urban-dwelling Black men with a smoking history to explore their experiences with and attitudes toward lung health promotion activities. Specifically, we examined their tobacco use history, smoking cessation experiences, and knowledge and attitudes related to lung cancer early detection screening. Supplemental survey data on the same topics were collected to support qualitative findings.

## 2. Materials and methods

### 2.1 Study design

The study used a descriptive qualitative study design [28]. Data were collected as part of a larger lung health promotion intervention development study for Black men [29, 30]. The study partner was a federally qualified healthcare center (FQHC) affiliated with a large academic medical center in Chicago. This study’s recruitment and data collection occurred within three months (June–August 2021), and the qualitative study participants were not enrolled in other portions of the study.

### 2.2 Theoretical framework

Andersen’s Behavioral Model of Health Services Utilization [31] guided the conduct of the study. Andersen’s Model is a comprehensive framework that explains the various factors influencing individuals’ engagement in health-related behaviors, including smoking cessation and lung cancer screening. The first is predisposing factors, the individual characteristics that affect one’s inclination towards health services utilization. In the context of smoking cessation and lung cancer screening, predisposing factors would include demographics (age, gender, education) and health beliefs (perceived susceptibility to lung cancer, perceived severity of smoking-related health risks). Enabling factors refer to the resources and opportunities that facilitate or hinder health service utilization. Regarding cessation and screening, enabling factors might involve access to healthcare services (availability, affordability), health insurance coverage, and proximity to screening facilities. Finally, factors relate to an individual’s perceived and evaluated need for healthcare services. In the case of smoking cessation and lung cancer screening, need factors include perceived health status, symptoms and risks associated with smoking. Overall, the model suggests that a combination of awareness, access and perceived need will determine an individual’s likelihood of engaging in smoking cessation and lung cancer screening behaviors. This framework provides a structured way to understand and predict these health-related behaviors by considering multiple influencing factors.

### 2.3 Recruitment and enrollment

This study used community and clinic-based recruitment strategies to identify and enroll eligible Black men. The study inclusion criteria were: (1) self-identity as a Black male, (2) current smoker or have quit in the last ten years, (3) no history of lung cancer, and (4) ability to provide informed consent. Community-based recruitment activities included posted flyers with study details at community venues (*i.e.*, barbershops and churches) frequented by Black men and *via* social network connections (*i.e.*, word of mouth). In addition, the electronic health record (EHR) system was also used to identify potential participants at the FQHC’s appointment list, which was scanned daily for eligible men. Once potential participants arrived for their appointments, clinic staff asked them if they would be interested in hearing about a research study. If the participants expressed interest, they were directed to speak to the project research assistant, who described the purpose of the study, assessed eligibility and obtained informed consent. Recruitment and enrollment of participants occurred continuously. All study participants received $40 as compensation for their participation.

### 2.4 Study procedures

Recruitment and enrollment of participants occurred continuously. The semi-structured in-depth interviews were held in person or over the phone due to restrictions associated with the COVID-19 pandemic. Out of 25 patients, 15 completed the interview in person and ten over the phone. The interviews took 30–45 minutes. As participants arrived for each scheduled interview, research staff members obtained written informed consent, and participants completed a brief (5–10 minutes) survey. Those individuals interviewed over the phone signed a written consent form *via* a secure email link. The survey was meant to supplement qualitative findings and measured demographics, smoking behaviors and knowledge and attitudes regarding lung cancer and cancer screening. Demographic variables measured included age, race/ethnicity, gender identity, education and health status. Standard smoking questions measured include current smoking status, age of smoking initiation, frequency and quantity of smoking, time to the first cigarette after waking up, type of cigarette smoked (mentholated *vs.* non-mentholated), and use of other tobacco-containing products, including e-cigarettes [32]. Questions related to lung cancer knowledge and attitudes included the prevention of lung cancer, risk perception, worry related to the development of lung cancer, and the durability of lung cancer was adapted from a study conducted by Jonnalagadda and colleagues [23]. Lung cancer screening questions were adapted from prior surveys and included awareness of LDCT, prior history of screening, interest in screening and reasons for considering screening [33–35]. All surveys were completed in person or *via* Redcap, a secure online data collection tool.

Established qualitative methodology—trained moderators, using a moderator’s guide, the audio recording of interviews, and immediate post-session facilitator debriefing—were used [36, 37]. The moderator’s guide was developed based on the existing literature on tobacco use and lung cancer screening among Black smokers [38, 39]. The moderator’s guide covered the following domains relevant to understanding the experiences and opinions of Black men related to lung health promotion: history of tobacco use, smoking and health knowledge, provider communication about smoking, knowledge of symptoms and risk factors for lung cancer, and knowledge and attitudes regarding lung cancer screening.

### 2.5 Data analysis

A total of 25 Black male smokers completed the in-depth interviews. Descriptive statistics (frequencies, percentages, means and standard deviations (SD)) were used to characterize study participants’ demographic characteristics, smoking behaviors, and attitudes regarding lung cancer and lung cancer screening from the quantitative survey administered before the in-depth interview. All in-depth interviews were audio-recorded and verbatim transcripts were created. The qualitative interview data were analyzed using deductive thematic analysis [40]. The deductive method of thematic analysis involves approaching the data analysis with pre-determined themes and categories, according to which the data is evaluated [41]. The study codebook consisted of a priori codes, derived from the existing literature and the moderator’s guide, and emergent codes, which were identified as the analyses progressed. Each author reviewed the codes, categories and themes in an iterative process; meetings were held to discuss and document analytic insights, assumptions and decisions. Thematic saturation was assessed and achieved with the final sample of 25 participants [42]. Thematic saturation was determined when successive interviews and data analysis consistently revealed redundant themes without introducing new significant insights, signifying the attainment of thematic saturation. This was corroborated through regular comparison and reflection on existing themes, supported by the collective judgment of the research team.

## 3. Results

### 3.1 Quantitative results

Table 1 displays the demographic characteristics of study participants (N = 25). All participants were Black males with a mean age of 57.5 years. Sixty-four percent of participants had a high school education or higher. Most participants rated their health status as fair (56%). Table 2 summarizes the participant’s smoking history. Eighty-four percent of participants were current smokers. Most participants (92%) smoked 6–7 days per week, with the modal category of the number of cigarettes smoked as 6–10 a day (48%). Of the current smokers, 80% reported smoking within the first 30 minutes after waking up (an indicator of high nicotine dependency). Nearly all study participants (96%) reported currently or previously smoking (among former smokers) a mentholated tobacco brand. Poly-tobacco use (using one or more types of nicotine-containing products) was also common among participants (66%). Fifty-six percent of participants reported making a quit attempt in the past twelve months.

Table 3 summarizes the survey data regarding attitudes toward lung cancer and screening. More than half of the participants (52%) believed lung cancer could be cured. The perceived risk for lung cancer was mixed, with 56% of participants endorsing that they considered themselves moderate or high risk for lung cancer and the remaining 44% at no or low risk. Although 40% of participants reported having had a test to check their lungs for cancer, only 24% reported having heard of an LDCT for lung cancer screening. Forty percent of participants indicated that they “often” worried about getting lung cancer, and 84% reported that they would consider receiving LDCT if they were eligible for the test. The primary reasons participants indicated they would get an LDCT lung cancer screening exam were to “have peace of mind” (40%) and to “find lung cancer early” (32%).

### 3.2 Qualitative results

Table 4 includes a summary of the key qualitative findings related to the domains of interest, including (1) tobacco use history, (2) smoking and health, (3) receipt of smoking cessation resources from providers, (4) lung cancer knowledge, (5) knowledge and attitudes about lung cancer screening, (6) questions related to screening, (7) reasons to be screened, and (8) perceived barriers to screening. Below we describe each primary theme and subthemes with illustrative quotations, as appropriate.

#### 3.2.1 Tobacco use history

Most study participants reported starting to smoke in their mid-teens. Participants explained the onset of smoking initiation, including peer pressure, wanting to fit into their peer groups, and imitating the behaviors of older peers and adults.

“I used to hang around older guys. So, they were smoking, I was smoking.” (P.4)

“All my friends were smoking, so I wanted to join in.” (P.9)

“Why? Because everyone, all the cool guys, was doing it.” (P.20)

“We drank to be noticed, we smoked to be noticed, we wanted to be older than what we were.” (P.23)

Easy access to cigarettes and early exposure to adult smoking were discussed as additional contributing factors to initiating tobacco at an early age. Ease of access was linked to the low price of cigarettes, being sent to buy cigarettes for adults, and the ability to steal them from adult smokers. Exposure to adult smokers increased the normalization of smoking as an acceptable behavior.

“They sell cigarettes out in the streets for a dollar a piece. 75 cents apiece.” (P.12)

“My friend’s parents smoked. They used to send us to the store to get cigarettes.” (P.22)

“When I first smoked, it was Viceroys because that’s what my father smoked. I used to sneak in and steal his cigarettes.” (P.25)

“My mama and father used to smoke, so I wanted to smoke. I thought I could be grown.” (P.12)

Consistent with the extant literature, most participants smoked a mentholated cigarette brand [43]. Participants described their reasons for selecting a mentholated brand. Responses included selecting a specific cigarette brand (such as Newport) because of its popularity within the Black community. Others discussed targeting by the tobacco industry as responsible for the high prevalence of menthol use in Black communities. Still, others discussed the sensory experiences of smoking a menthol compared to a non-menthol cigarette. One individual reported the incorrect belief that smoking mentholated cigarettes are a healthier option.

“Yeah. Menthol gives you more of a powerful feeling. Makes you enjoy it more.” (P.3)

“Newport is one of the number one top cigarettes in America.” (P.12)

“And then you know how the cigarette companies, the tobacco industry, how they market things and how they market in the Black community.” (P.16)

“Because at that point, they were talking about lung cancer. Well, I took under consideration—if you smoke menthol, you won’t get cancer.” (P.23)

#### 3.2.2 Concerns about smoking and health

Although researchers have linked smoking to various health-related conditions, participants focused exclusively on lung cancer when assessing the health risks associated with smoking. The level of concern and perceived risk for lung cancer was mixed among study participants, ranging from an elevated level of concern to no perceived risk. Elevated levels of concern and perceived risk for lung cancer were primarily associated with a history of long-term smoking and exposure to a family member diagnosed with lung cancer.

“I think I have a high risk, because I’ve been smoking for so long, since I was 17.” (P.1)

“Very [concerned]. I don’t want lung cancer.” (P.2)

“I’m very concerned. Like, every time I light one, I’m pretty concerned.” (P.3)

“I’m seriously considering stopping because like I said previously, my father’s brother, he died from lung cancer. So, that’s something I don’t wanna bring on myself.” (P.19)

However, not all participants considered themselves at risk for lung cancer. Individuals who smoke fewer cigarettes per day or who have recently cut down on the number of cigarettes smoked were more likely to consider themselves to be at a lower risk for the development of lung cancer.

“Because I didn’t think it was that serious. Six cigarettes in a day? Would that affect me? We can be our own doctor sometime, and I said ‘Naw, that ain’t gonna affect me’. I know people smoking two or three packs a day.” (P.23)

“Right now, mine [lung cancer risk] would be medium because I have slowed down a lot. I don’t smoke as many cigarettes a day as I used to. Right now, I just smoke maybe 10 to 12 cigarettes a day.” (P.25)

#### 3.2.3 Receipt of smoking cessation resources from providers

Healthcare providers are essential in educating patients about the health risks associated with smoking and providing cessation treatments. Based on the survey data from study participants, eighteen participants reported that their healthcare providers asked about their smoking and provided information about the risks of smoking-related illnesses such as respiratory problems, hypertension, heart disease, oral cancer and lung cancer.

“The doctor says you’ve got to stop smoking because you’re going to mess your lungs.” (P.13)

“Dentist said quit smoking ‘cause you’re gonna stain up your teeth, you know? And probably catch lung cancer.’ They all encourage people to stop smoking. They always did me.” (P.22)

In addition to encouragement to quit smoking, some participants also reported receiving resources from their healthcare providers to aid with cessation efforts. Provider resources were primarily related to nicotine replacement therapies such as the patch. Among patients not ready to make a quit attempt, some providers were reported to have offered future assistance with quitting.

“Oh yeah, definitely. They always offered me patches and stuff.” (P.24)

“They told me I should take the patch. Put the patch on every day.” (P.9)

“They ask me about nicotine patches and all this stuff. She even prescribed me some nicotine stuff and all that.” (P.18)

“Well, they let me know that once I decide that I’m ready, all of those tools to help me stop would be provided and that they would help.” (P.19)

Consistent with the literature, participants’ reactions to nicotine replacement therapies as part of smoking cessation efforts were mixed [44]. One participant reported trying the patch but discontinuing use because of a perceived lack of benefit. Others reported being prescribed patches but not using them and instead making a “cold turkey” quit attempt.

“No. I didn’t even take the patches. I got them, but I didn’t use them.” (P.2)

“I tried them [patches], but it actually done no good.” (P.12)

“No [did not use the patches]. I quit cold turkey.” (P.24)

Alternatively, several participants indicated that their provider recommended quitting but did not offer specific advice or resources. Other participants reported not receiving advice or resources for cessation. One participant questioned the level of support provided to patients from other racial/ethnic backgrounds and attributed the lack of assistance with cessation as resulting from racism.

“Not in depth; he just asked me did I smoke, and I said, ‘Yeah.’ ‘How long?’ You know, 30, 40 years. ‘Stop smoking!’ That’s it.” (P.7)

“No, I haven’t—she hasn’t really said nothing about why I should stop smoking.” (P.16)

“They never mention it. No leads to no medication, or groups, or anything like that.” (P.3)

“Now, I don’t know because I was a Black man, I don’t know what he woulda said to the White man, or the Italian man, or whatever nationality. The only thing I got was a booklet. No explanation at all, just a booklet. Because of my skin, right.” (P.23)

#### 3.2.4 Lung cancer knowledge

Subthemes related to knowledge about lung cancer included general information, signs and symptoms, and factors related to the causes of lung cancer. In general, knowledge about lung cancer was limited. However, study participants were aware of the primary symptoms of lung cancer, including coughing, shortness of breath, chest pain, and weight loss. Some individuals’ knowledge about lung cancer was informed by knowing someone who had been diagnosed with it. Others reported obtaining information from commercials that described the link between smoking and lung cancer.

“Not very much. I know it’s cancer. That’s about all I really know.” (P.9)

“Dry cough, throwing up blood, difficulty breathing. Those are the things that I know about it.” (P.24)

“Chest pains. Breathing problems. Change in activities.” (P.6)

“I’ve seen a few people with lung cancer who looked pretty bad. They lose weight. They couldn’t breathe on their own.” (P.3)

“They run a lot of commercials [on television] about lung cancer and smoking. So, I pay attention to that.” (P.8)

Study participants correctly identified several factors related to the development of lung cancer. For example, smoking-related factors contributing to lung cancer were correctly identified as cigarette smoking and exposure to secondhand smoke. Genetic factors were also understood as contributing to lung cancer risk. Finally, environmental factors, such as occupational risks were named as risk factors for lung cancer.

“Cigarette smoking can lead to and cause lung cancer.” (P.18)

“…and hereditary—genetic disposition.” (P.19)

“Secondhand smoke.” (P.23)

“You can be working in a chemical environment at work and steel mills, and you can get lung cancer.” (P.24)

#### 3.2.5 Knowledge and attitudes about lung cancer screening

Only six study participants had heard about an LDCT test for lung cancer screening, but none understood the specifics of the screening process. To understand initial attitudes about screening, the moderator briefly described LDCT to all participants, including the screening process, eligibility, costs, and the risks and benefits of testing. Initial reactions to the availability of a test for lung cancer early detection were positive.

“I need it. I need it. It sounds good for my health. It sounds good to know.” (P.2)

“I would definitely do it. I would want to, if I do have anything, I would want to catch it early. I do want to live. I want to live. Momma, I want to live.” (P.10)

“If it [LDCT scan] can help detect early stages of cancer, I’m all for it.” (P.19)

#### 3.2.6 Questions related to screening

After explaining screening and gauging initial attitudes and interests, participants were asked to indicate specific screening questions. In summary, the questions asked were associated with the screening logistics (how long the screening will take, when results will be received, the need for pre-authorization), costs and insurance coverage, health risks and side effects associated with screening, and communication about and treatment planning associated with any abnormal findings.

“Oh, just how long we will it [the test] take. And how long will it be before I get the results back? And do I have to get authorization from my insurance.” (P.3)

“What is the test gonna determine and will I get the results the same day?” (P.14)

“What’s the side effects? I’d ask them, ‘is this dangerous?’ That’s all.” (P.15)

“And then, what are some of the steps being done to correct whatever problems that might be found?” (P.20)

#### 3.2.7 Reasons to be screened

Participants voiced several reasons for obtaining screening. The most prevalent among the responses was a desire to know if smoking has damaged their lungs. In addition, participants experiencing physical symptoms expressed a desire to determine if those symptoms are associated with a more significant health concern. Finally, participants discussed the perceived benefits of early detection of lung cancer as a reason for participating in screening.

“Like, the flight of stairs that I used to not breathe so hard getting up, I breathe a little bit harder now. So, I would take one of those x-rays to see if this just me being old, or just the cigarette smoking… I would want to check it out.” (P.3)

“If it can help detect early stages of cancer, I’m all for it.” (P.19)

“The one thing is to find out what damage have I done to my lungs?” (P.23)

Although not a typical response, two individuals indicated that participating in lung cancer screening would motivate quitting smoking.

“I can’t think of anything that would stop me, being that I’m a smoker, I’ve been abusing my body by smoking, and I would like to change my behavior, which encouraged me to go through this process [being interviewed for the study]. So, me wanting to change, I have no reason not to go this route [getting screened].” (P.14)

“The reason why I would think about it? It would give me a reason to stop if I were told that my lungs were damaged.” (P.22)

#### 3.2.8 Perceived barriers to screening

Although all participants had positive attitudes about LDCT screening, many were not interested in screening for themselves. Participants were asked to describe barriers to receipt of lung cancer screening for themselves or the larger Black community. At the personal level, reasons for the lack of interest in screening varied and included not wanting to know if something was wrong, a philosophy of “if it ain’t broke, don’t fix it”, and a lack of perceived risk associated with lung cancer. Other personal barriers include cost, fear and logistical barriers like work schedules. Comments reflecting these themes are as follows:

“I mean, if I take it, I’m going to want to know if my insurance covers it, because I don’t have to pay out of pocket, of course.” (P.1)

“I work a lot. So, it depends on the day and the time.” (P.8)

“Sometime man we don’t wanna know what’s wrong with us. I don’t wanna know that I’m fucked up. That I’m messed up, because now I got to do something about it.” (P.7)

“Fear. Fear that you might have something sometimes makes you not want to get a test. Some people, believe it or not, some people would rather not know than know.” (P.10)

“I’m not interested. And I gotta tell you the reason. I guess my attitude toward life is; if it’s not broken, don’t fix it. And then who wants to hear bad news?” (P.18)

At the community level, lack of information and access to health care were identified as primary barriers to widespread screening among Black smokers.

“I just don’t think there’s enough information this coming to the community about lung cancer for them to understand that they should get checked about it.” (P.21)

“This community lacks resources and medical care, so they don’t get the opportunity to go to the doctor like they want to go.” (P.21)

## 4. Discussion

This study examined Black male smokers’ experiences and attitudes associated with lung health promotion. Given the known smoking related health disparities observed among Black men who smoke, additional information is needed to inform the development of combined lung cancer screening and smoking cessation interventions for this underserved population of smokers. Participants in our sample were typically light smokers, smoked a mentholated brand of cigarette, were highly nicotine dependent, had engaged in a recent quit attempt, and had a diagnosis of a chronic health condition exacerbated by smoking. The smoking profile of participants in this study was consistent with the extant literature on smoking among Black adults which document a population that is highly addicted with recent and unsuccessful quit attempts [45–47]. These findings underscore the importance of continued efforts to improve access and effectiveness of smoking cessation interventions for Black smokers. Although knowledge about lung cancer was limited, study participants understood the link between smoking and lung cancer. Indeed, more than half of the study participants expressed some level of worry about developing lung cancer and perceived themselves to be at moderate to high risk for the development of lung cancer.

Many participants reported attempting to quit or reduce their smoking in the previous 12 months. However, barriers to smoking cessation were reported. First, study participants underscored the extant literature on the reduced likelihood of healthcare providers providing direct support for smoking cessation to Black smokers [48]. Further, our findings were consistent with the literature suggesting a reluctance among Black smokers to use evidenced-based treatments such as nicotine replacement therapies when prescribed [39, 44, 49]. However, study findings suggest potential promise for increasing access to smoking cessation treatments for Black men because interest in smoking cessation was high. Several modifiable barriers to engagement in smoking cessation (provider behaviors) and improving smoking cessation outcomes were identified.

In addition to the prevention of lung cancer *via* the reduction of smoking, early detection is the second cornerstone of improving lung health inequalities. With the advent of LDCT lung cancer screening, reducing lung cancer mortality among high-risk smokers has become a reality [50]. However, for LDCT to achieve the maximum public health benefit, interventions to increase the awareness and uptake of lung cancer screening among smokers in the general population and groups of smokers with known barriers to healthcare access are warranted [29, 51–54]. Consistent with the extant literature [55–58], our study participants’ knowledge and awareness of LDCT lung cancer screening was minimal. Due to a limited understanding of lung cancer screening, participants were provided a brief description of the process to gauge interest, attitudes and potential concerns. Initial reactions to the availability of a practical test for early lung cancer detection were positive, with most participants indicating a willingness to be screened if eligible. However, not all individuals were interested in screening. As previously reported in the general cancer screening literature, fear and a reduced willingness to screen without symptoms were barriers among the men in our sample [59]. In addition to these barriers, study participants had multiple questions about the screening process that would need to be addressed before final screening decision-making. Participants’ questions were associated with numerous issues, including the process for obtaining screening, costs, health risks, side effects, and communication about and management of abnormal findings.

Given the known health inequalities associated with lung cancer, additional research is needed to understand better factors associated with engagement in lung health promotion interventions (smoking cessation and lung cancer screening) in high-risk groups. Information relevant to developing lung health promotion interventions among Black male smokers remains limited to date. This study reveals the barriers and opportunities to address lung health promotion for Black men who use tobacco products.

## 5. Implications for lung health promotion interventions

Study findings point to several issues pertinent to lung health promotion interventions in this population. The first is the need to develop strategies for increasing access to smoking cessation treatments, especially among patients with access to primary care services. Provider-led interventions have demonstrated effectiveness in supporting smoking cessation activities [60]; however, the consistency of provider-based interventions, including Ask, Advise and Refer, remains a significant barrier to treatment provision [61]. Provider time constraints and lack of training are commonly reported barriers to offering patients smoking cessation treatments [61]. Additional research is needed to identify strategies to overcome these barriers to realize the benefits of clinic-based and provider-facilitated smoking cessation treatments. Further, emerging best practice in lung cancer screening suggests the importance of offering combined lung cancer screening and smoking cessation services [62, 63]. As these practice models emerge, cross-consideration of health promotion activities should be included in all services developed to assist with smoking cessation or engage patients in lung cancer screening.

Although participants reported a desire to quit smoking, utilizing evidence-based treatments such as nicotine replacement therapies represents a significant barrier to successful quitting in this group. The importance of nicotine replacement therapies for achieving smoking cessation is essential for Black smokers who are likelier to smoke a mentholated cigarette brand. Research has shown that menthol smokers have greater difficulty achieving abstinence [64, 65]. Given the data showing increased difficulty with cessation among Black menthol smokers [66] and a reluctance to use nicotine replacement therapies as part of treatment [67], additional research is needed to understand such correlations to inform more tailored treatment options for Black men. Increasing education about the efficacy of pharmacological approaches and increasing access to evidence-based treatments may help to improve cessation outcomes among Black male smokers.

Finally, study participants expressed receptivity to participating in lung cancer early-detection screening. Many study participants (84%) indicated that they would be interested in receiving lung cancer screening if eligible. Interest in screening was related to a desire to know if smoking had negatively impacted their lungs and an understanding of the benefits of early detection. However, several participants had questions about screening that warrant additional education. The development of population-level lung cancer screening education and intervention approaches is in its infancy. Effective means to provide knowledge around lung cancer screening and support for screening decision-making require further research.

Culturally targeted health promotion interventions for lung cancer screening may have benefits for improving screening and early detection in Black communities [29, 68]. Targeted interventions can improve behavioral outcomes when there is an adequate understanding of the drivers of health risk behaviors in specific population groups and the mechanisms that influence those behaviors [69]. Emotional barriers such as fear of cancer and fatalism associated with a cancer diagnosis are powerful forces in preventing cancer screening among Black patients [70]. Shared decision-making has shown to be an effective tool for engaging participants [71, 72], and fear and uncertainty could be dealt with through shared knowledge and alternatives. Research is needed to determine the differential impact of standard versus culturally targeted interventions to increase lung health promotion among Black male smokers [30].

Barriers to participating in LDCT, including concerns about costs and logistical barriers such as work schedules, are known as social determinants of health (SDOH) [73]. Many community health clinics have adopted SDOH screening policies and patient navigation care models. Patient navigators may assess SDOH needs and barriers and provide resources to address them [74]. However, the current reimbursement landscape does not facilitate the effort to improve LDCT participation. Meaningful payment reforms are necessary for SDOH screening to reduce logistical barriers to LDCT participation. In addition, given the severe patterns of racial residential segregation, expanding healthcare facilities with cancer screening capacity in highly segregated and economically disadvantaged communities can reduce barriers associated with access.

## 6. Limitations

The study findings should be viewed in the context of a few limitations. While in-depth interviews are an excellent exploratory method to identify essential constructs and intervention targets, extending these findings to develop large-scale data collection methods, including survey research methods, would be helpful. Although appropriate for qualitative studies, our study included a small sample of the target population from a single geographical location; thus, additional studies are required. Generalizability was not the goal of this qualitative research; however, Black male smokers from other geographical locations may have other opinions or experiences germane to understanding the development of lung health promotion interventions in this population. Further, the sample may not fully represent the broader underserved population. This is evident from the fact that 84% of the study population has health insurance, and 64% have at least a high school education.

Although a brief quantitative survey was used to capture preliminary attitudes regarding screening, due to the scant literature in this area, a more exhaustive assessment of lung cancer knowledge and screening knowledge and attitudes is needed. We collected survey data that, in theory, could have been used to calculate pack years. However, because the survey used categorical response options, we were unable to gather precise information about the length of time smoked, the number of days smoked, and the number of cigarettes smoked needed for an accurate pack year calculation. Finally, the experiences of Black men were the particular focus of this study. However, future studies should also examine the experiences and perspectives of Black women who smoke.

## 7. Conclusions

The optimal strategy for engaging patients with and delivering smoking cessation as part of LDCT screening remains unclear [62]. In addition, few studies have sought to engage Black male smokers who are disproportionately at risk for lung cancer incidence and mortality [30]. Developing effective lung health promotion interventions for Black male smokers is crucial in eradicating smoking-related health inequities in this underserved population. Combined, the information obtained from this study has implications for developing multi-level health promotion interventions targeting smoking cessation and lung cancer screening among Black men.

## Figures and Tables

**TABLE 1.** Demographic characteristics of study participants (N = 25).

Variables	N	%
Age		
Mean = 57.5 years (range 45–71)		
Race		
Black	25	100%
Gender		
Male	25	100%
Education		
Less than high school	9	36%
High school, GED*, or Trade School	6	24%
Some College	9	36%
Bachelor’s degree	1	4%
Health insurance		
Insured	21	84%
Uninsured	4	16%
Health Status		
Very good	5	20%
Good	5	20%
Fair	14	56%
Poor	1	14%
Having a primary care provider		
Yes	17	68%
No	8	32%

GED*: General Educational Development.

**TABLE 2.** A Description of the smoking behaviors and experiences of study participants (N = 25).

	N	%
Current smoker		
Yes	21	84.0
No	4	16.0
Days of smoking per week		
2–3 days	1	4.0
4–5 days	1	4.0
6–7 days	23	92.0
Numbers of cigarette smoking per day		
0–10	15	60.0
11–20	7	28.0
21 and over	3	12.0
Time to start first cigarettes after waking up		
Within 5 minutes	9	36.0
5–30 minutes	11	44.0
31–60 minutes	1	4.0
>60 minutes	4	16.0
Type of cigarettes do you usually smoke		
Menthol	24	96.0
Both regular and menthol	1	4.0
Ages when started smoking		
6–10 years old	2	8.0
11–15 years old	13	52.0
16–20 years old	6	24.0
21 years and older	4	16.0
Other forms of smoking		
Cigars	9	36.0
Electronic or e-Cigarettes	1	4.0
Other	4	16.0
None of the above	11	44.0
Provider knows a smoker		
Yes	21	84.0
No/Not applicable	4	16.0
Provider advised a quit attempt		
Yes	18	72.0
No	3	12.0
Not applicable	4	16.0
The provider offered resources to quit		
Yes	13	52.0
No	10	40.0
Missing	2	8.0
Diagnosed with a smoking-related illness		
Yes	4	16.0
No	20	80.0
Do not know	1	4.0
Diagnosed with a chronic illness made worse by smoking		
Yes	14	56.0
No	11	44.0
Made a quit attempt in the past 12 months		
Yes	12	48.0
No, but cut down	3	12.0
No cessation efforts	7	28.0
N/A Former smoker		

N/A: Not applicable.

**TABLE 3.** Attitudes about lung cancer and lung cancer screening (N = 25).

	N	%
1. Perceived risk for lung cancer		
Not at all	2	8.0
Low risk	9	36.0
Moderate risk	7	28.0
High risk	7	28.0
2. Level of worry about lung cancer		
Often	10	40.0
Sometimes	6	24.0
Not at all	9	36.0
3. Can lung cancer be prevented		
Yes	13	52.0
No	3	12.0
Do not know/Not sure	9	36.0
4. Can lung cancer be cured		
Yes	15	60.0
No	2	8.0
Do not know/Not sure	7	28.0
5. Have you ever had a test to check your lungs		
Yes	10	40.0
No	14	56.0
Do not know/Not sure	1	4.0
6. Have you ever heard of an LDCT scan		
Yes	6	24.0
No	18	72.0
Do not know/Not sure	1	4.0
7. Would you consider taking it if eligible		
Yes	21	84.0
No	1	4.0
Do not know/Not sure	2	8.0
8. Reasons for screening		
1. Having a lung scan will help find lung cancer early	8	32.0
2. Having a lung scan will lower my chances of dying from lung cancer	4	16.0
3. Having lung cancer screening will give me peace of mind	10	40.0
9. Reasons for not screening		
1. I worry about finding something wrong	4	16.0
2. I worry about exposure to radiation or damage to my lungs	0	0
3. I don’t have a regular doctor to schedule one for me	0	0
4. I don’t have insurance, so I worry about the cost of the test	1	4.0
5. I don’t have any lung problems or symptoms	2	8.0
6. I don’t know enough about the test to feel comfortable	1	4.0
7. I would rather NOT know if I have any lung problems	1	4.0
8. I worry about being blamed for having smoked	1	4.0
9. I’m not at high risk for lung cancer	2	8.0

LDCT: low-dose helical computed tomography.

**TABLE 4.** Summary of qualitative findings.

Main themes	Subthemes	Qualitative findings	Example quotations
1. Tobacco use history			
	1.1 Age of starting smoking and reasons for smoking	Participants started smoking in their mid-teens. Several reasons for smoking initiation were explained, such as peer pressure.	“All my friends were smoking, so I wanted to join in.” (P. 9)
	1.2 Contributing factors to start smoking	Easy access to cigarettes and early exposure to adult smoking were identified.	“They sell cigarettes out in the streets for a dollar a piece. 75 cents apiece.” (P. 12)
	1.3 Cigarette brands used and reasons	Participants preferred to use mentholated cigarette brands for several reasons, such as selecting a popular specific brand among the Black community.	“Newport is one of the number one top cigarettes in America.” (P. 12)
2. Concerns about smoking and health	2.1 Level of concern and perceived risk of smoking-related health issues	Mixed levels of concern and perceived risks of smoking-related health ranged from low to high.	“I think I have a high risk, because I’ve been smoking for so long, since I was 17.” (P. 1) “Because I didn’t think it was that serious. Six cigarettes in a day? Would that affect me?” (P. 23)
3. Receipt of smoking cessation resources from providers			
	3.1 Receiving information about smoking-related illness	Most of the participants received information about smoking-related illnesses from their healthcare providers.	“The doctor says you’ve got to stop smoking because you’re going to mess your lungs...” (P. 13)
	3.2 Receiving specific recourses to aid smoking cessation	Resources included prescribing nicotine patches.	“They ask me about nicotine patches and all this stuff. She even prescribed me for some nicotine stuff and all that.” (P. 18)
	3.3 Reactions to the use of nicotine replacement therapies	The reaction to using nicotine replacement therapies was mixed; for example, perceived lack of benefit and using “a cold turkey” quitting method.	“I tried them [patches], but it actually done no good.” (P. 12) “No [did not use the patches]. I quit cold turkey.” (P. 24)
	3.4 Perceived level of support	Some participants perceived that Black smokers do not receive treatment at the same level as other races.	“Now, I don’t know because I was a Black man, I don’t know what he woulda said to the white man, or the Italian man, or whatever nationality. The only thing I got was a booklet. No explanation at all, just a booklet. Because of my skin, right.” (P. 23)
4. Lung cancer knowledge			
	4.1 General knowledge	Knowledge about lung cancer was limited.	“Not very much. I know it’s cancer. That’s about all I really know.” (P. 9)
	4.2 Signs and symptoms of lung cancer	Insufficient knowledge about the signs and symptoms of lung cancer.	“Dry cough, throwing up blood, difficulty breathing. Those are the things that I know about it.” (P. 24)
	4.3 Factors causing lung cancer	Several factors were identified, including cigarette smoking, secondhand smokers, and genetic and environmental factors.	“Cigarette smoking can lead to and cause lung cancer...” P. 18 “Secondhand smoke.” (P. 23)
5. Knowledge and attitudes about lung cancer screening	Knowledge and attitudes toward LDCT lung cancer screening	Few participants have heard about LDCT screening, but none understood the LDCT screening process. After the moderator briefly described LDCT, participants had more positive attitudes about LDCT.	“I would definitely do it. I would want to if I do have anything, I wouldwant to catch it early. I do want to live. I want to live. Momma, I want to live.” (P. 10) “If it [LDCT scan] can help detect early stages of cancer, I’m all for it.” (P. 19)
6. Questions related to screening	Specific questions related to LDCT screening	Specific questions included the screening process, costs and insurance coverage, side effects related to the screening, and abnormal results.	“Oh, just how long we will take it. And how long will it be before I get the results back? And do I have to get authorization from my insurance?” (P. 3)
7. Reasons to be screened	Reasons for considering getting the screening	Several reasons were identified for receiving lung cancer screening, such as lung health status, early detection of lung cancer, and motivation for quitting smoking.	“...because if it can help detect early stages of cancer, ... I’m all for it.” (P. 19) “The reason I would think about it? It would give me a reason to stop if I were told that my lungs were damaged.” (P. 22)
8. Perceived barriers to screening	Barriers affecting getting the screening	Several barriers that affect deciding to get lung screening were identified at personal and community levels.	“I mean, quite surely. I mean, if I take it, I’m going to want to know if my insurance covers it because I don’t have to pay out of pocket, of course.” (P. 1) “I just don’t think there’s enough information this coming to the community about lung cancer for them to understand that they should get checked about it.” (P. 21)

LDCT: low-dose helical computed tomography.

## Data Availability

The data presented in this study are available on reasonable request from the corresponding author.
